# Cooperation of TGF‐β and FGF signalling pathways in skin development

**DOI:** 10.1111/cpr.13489

**Published:** 2023-05-07

**Authors:** Xinxin Li, Rongfang Xie, Yilin Luo, Runlu Shi, Yuanqiang Ling, Xiaojing Zhao, Xuejuan Xu, Weiwei Chu, Xusheng Wang

**Affiliations:** ^1^ School of Pharmaceutical Sciences (Shenzhen) Sun Yat‐Sen University Shenzhen China; ^2^ Institute of Biopharmaceutical and Health Engineering (iBHE), Shenzhen International Graduate School Tsinghua University Shenzhen China; ^3^ Guangzhou Wishing Tree Hair Medical Technology Limited Company Guangzhou China; ^4^ Department of Endocrinology The First People's Hospital of Foshan Foshan China

## Abstract

The skin is a multi‐layered structure composed of the epidermis, dermis and hypodermis. The epidermis originates entirely from the ectoderm, whereas the dermis originates from various germ layers depending on its anatomical location; thus, there are different developmental patterns of the skin. Although the regulatory mechanisms of epidermal formation are well understood, mechanisms regulating dermis development are not clear owing to the complex origin. It has been shown that several morphogenetic pathways regulate dermis development. Of these, transforming growth factor‐β (TGF‐β) and fibroblast growth factor (FGF) signalling pathways are the main modulators regulating skin cell induction, fate decision, migration and differentiation. Recently, the successful generation of human skin by modulating TGF‐β and FGF signals further demonstrated the irreplaceable roles of these pathways in skin regeneration. This review provides evidence of the role of TGF‐β and FGF signalling pathways in the development of different skin layers, especially the disparate dermis of different body regions. This review also provides new perspectives on the distinct developmental patterns of skin and explores new ideas for clinical applications in the future.

## Graphical abstract

Developmental process of skin components, especially in the disparate dermis formation located on different part of the body.

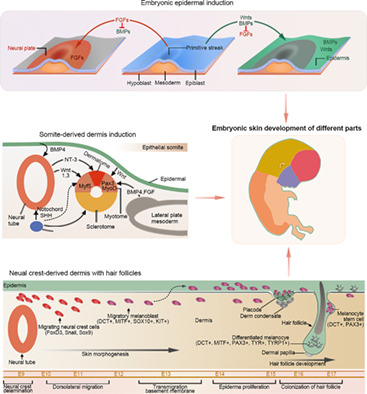

## 1 INTRODUCTION

Skin, the first barrier of the body, provides the function of protection, immunity and sensation.^1^ The mammalian skin is composed of the epidermis, dermis and hypodermis.^2^ The epidermis is a stratified squamous epithelium and is the outermost layer of the skin. Keratinocytes, melanocytes, Langerhans cells, Meckel cells and free nerve endings are present in the epidermis. This layer of the skin also contains derivative appendages, such as hair follicles (HFs). Beneath the epidermal layer is the dermis, which is composed of papillary fibroblasts, reticular fibroblasts, extracellular matrix and blood vessels that function together to nourish the avascular epidermis and maintain the structural integrity of the skin.^3^, ^4^ The hypodermis is the innermost layer of the skin tissue. It contains fat and connective tissue and mainly serves as an energy resource.

It is well known that the epidermis develops from the ectoderm. Compared to epidermis, dermal development is more complex since the dermis originates from various germ layers depending on its anatomical location. Lineage‐tracing studies have shown that the craniofacial dermis is derived from the neural crest cells (NCCs), which originate from the neural ectoderm, while the dorsal and ventral dermis originate from the paraxial mesoderm and lateral plate mesoderm, respectively.^5^, ^6^, ^7^ Based on the current understanding, different methods have been developed for producing skin or skin substitutes. However, a lack of understanding of dermal development contributes to various problems for producing skin in vitro. For example, the unknown identity of the regenerated skin and the loss of function due to the absence of appendages, all of which hinder the clinical use of in vitro regenerated skin. Therefore, there is an urgent need to understand skin development, particularly dermis development.

Recently, multi‐layered human skin organoids containing the epidermis, dermis and appendages (black HFs) have been successfully generated by exploiting the information of the face skin developmental pathway.^8^ During this process, only transforming growth factor‐β (TGF‐β) and fibroblast growth factor (FGF) signalling pathways were mediated at initial stages, which proved their importance in skin regeneration. It is widely known that TGF‐β and FGF family members are key regulators of skin morphogenesis. However, it is unclear whether these two signalling pathways are involved in the development of skin at other anatomical locations, and whether other signals are needed to determine the fate decisions during their involvement.

This review describes the functional roles and regulatory patterns of TGF‐β and FGF pathways at each stage during skin morphogenesis and provides details of the major developmental processes of the epidermis, including fate decision, epidermis stratification and HF and melanocyte generation. This review also describes the development of distinct dermis at different anatomical locations, such as the craniofacial dermis and dorsal and ventral dermis, a process that is not yet well understood. Our review provides new perspectives on skin repair and regeneration for basic research, as well as for specific clinical applications.

## 2 TGF‐β AND FGF SIGNALLING PATHWAYS

### 2.1 TGF‐β signalling pathway

The mammalian TGF‐β superfamily includes 33 genes that encode multiple functional cytokines.^9^ Among these cytokines, TGF‐β was first identified by A. B. Roberts et al. in the 1980s. Following this, the bone morphogenetic proteins (BMPs), activins, growth differentiation factors, müllerian inhibiting substance and nodals were discovered and were grouped into different TGF‐β subfamilies depending on their molecular identification or functions.^10^ In this section, TGF‐β and BMP subfamily members will be described in detail to highlight their pivotal role in regulating embryonic skin development.

**FIGURE 1:**
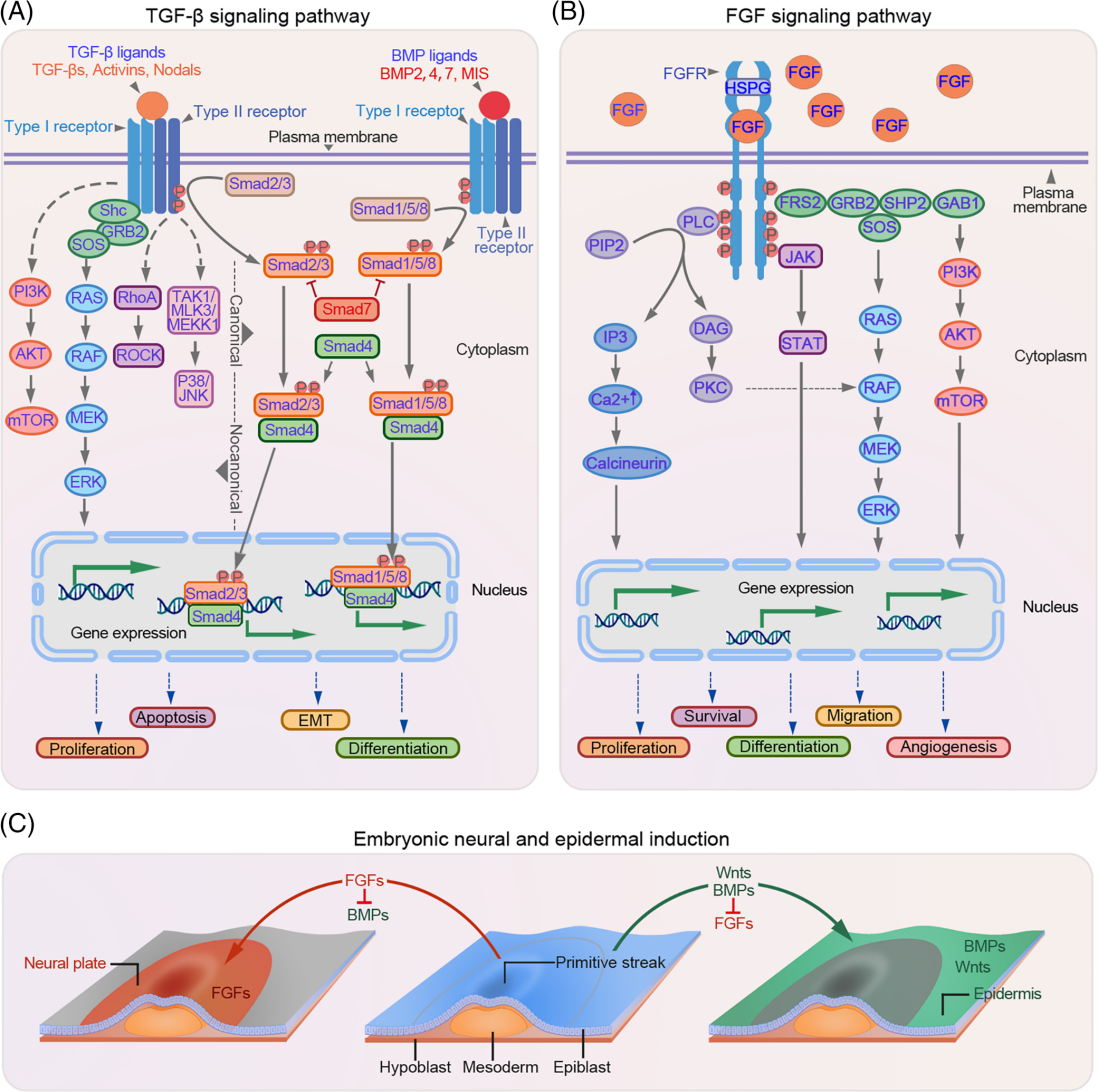
Illustration depicting the transforming growth factor‐β (TGF‐β) and fibroblast growth factor (FGF) signalling pathways. (A,B) Schematic representation of the TGF‐β and FGF signalling pathways. (C) Proposed signalling pathway for neural and epidermal induction in chick embryos. Medial epiblast cells of the gastrula express FGFs but not WNTs. FGFs suppress bone morphogenetic protein (BMP) expression, thus, inhibiting BMP signalling and promoting the neural fate of these epiblast cells. In contrast, lateral epiblast cells express both FGFs and WNTs. WNT signalling blocks the response of epiblast cells to FGFs, leading to the induction of BMPs. BMPs further suppress FGF expression and promote the epidermal fate of these lateral epiblast cells.

To date, three isoforms of TGF‐β and twenty functional BMP‐related proteins have been identified.^9^ These proteins are involved in various physiological processes, including cell proliferation and differentiation.^11^ TGF‐β and BMP ligands regulate target gene expression via both canonical (Smad‐dependent) and non‐canonical pathways (Smad‐independent). In the canonical pathway, TGF‐β/BMP ligands initiate the signal transduction cascade by forming a hetero‐tetrameric receptor complex with type I receptor components (TGF‐βRI/BMPR1A or BMPR1B) and type II receptor components (TGF‐βRII/BMPR2). Once the receptor complex is assembled, the activated type II receptors phosphorylate the serine residues present within the short glycine/serine‐rich motif (GS domain) of type I receptors. Subsequently, the phosphorylated type I receptor kinase is activated, which in turn phosphorylates its downstream targets, known as receptor‐regulated Smads (R‐Smads). In the case of TGF‐β, the R‐Smads mainly refer to Smad2 and Smad3, while in the BMP pathway, Smad1, Samd5 and Smad8/9 are the main R‐Smads. Activated R‐Smad proteins form a transcriptional complex with the co‐mediator Smad4 (co‐Smad), which is then translocated to the nucleus where it interacts with other transcription factors, corepressors or coactivators at the promoter to regulate gene expression, thereby mediating different biological effects. Smad7 in the TGF‐β pathway and Smad6/7 in the BMP pathway participate in the negative regulatory feedback and serve as inhibitors of R‐Smads (I‐Smads) by antagonising the phosphorylation of R‐Smads (Figure 1A).

### 2.2 FGF signalling pathway

Twenty‐three members of the FGF family have been identified and are divided into two groups. The secreted FGF proteins (FGF1–10 and FGF15–23) mediate signal transduction, whereas intracellular FGFs (FGF11–14) function in the nucleus by interacting with cellular effector proteins. For secreted FGFs, binding to the cell‐surface receptors is the first step in signal transduction.

Upon binding to the FGF receptors (FGFRs), FGFs activate four major signal transduction pathways, including RAS‐MAPK, PI3K‐AKT, PLCγ and STAT. The RAS‐MAPK pathway is initiated by the activation of the FGFR, which further interacts with its downstream target, FRS2a, leading to FRS2a phosphorylation and activation. Activated FRS2a binds to growth factor receptor‐bound 2 (GRB2) and subsequently recruits the guanine nucleotide exchange factor SOS, leading to RAS GTPase activation. The activated RAS GTPase further activates the downstream MAPK pathway. In addition, activated GRB2 can also bind to the adaptor protein GAB1, and this complex can activate the PI3K‐AKT pathway. The activation of the PLCγ and STAT pathways is dependent on the activation of PLCγ and STAT, respectively, by FGFRs (Figure 1B).

## 3 TGF‐β AND FGF SIGNALLING IN EPIDERMAL FORMATION, STRATIFICATION AND DIFFERENTIATION

Embryonic skin development begins with gastrulation, an early developmental process. During gastrulation, the embryo transforms from a one‐dimensional blastocyst to a multi‐layered gastrula containing the ectoderm, mesoderm and endoderm. The ectoderm can give rise to the surface ectoderm, which develops into the epidermis or neural ectoderm that forms the related neural components (Figure 1C). In the presence of specific signals, the epidermal fate is initiated and a multi‐potent basal layer arises, along with the formation of the basement membrane.^12^, ^13^ In mice, most basal progenitor cells proliferate symmetrically parallel to the basement membrane before E12.5, thereby producing daughter cells in a lateral layer to support the rapid growth of the embryo.^14^, ^15^ From E13.5, asymmetric division occurs and the fate of the proliferative basal progenitor cells changes to suprabasal cells.^13^, ^16^ During this period, the basal and suprabasal cells proliferate rapidly and begin to detach from the basal layer, marking the initiation of epidermis stratification.^17^ As stratification progresses, intermediate cells are periodically produced that differentiate into spinous cells. These intermediate cells further produce differentiated cells, enabling the establishment of a fully functional epidermis comprising basal, spinous, granular and cornified layers.^18^

BMP4 is the first reported and generally accepted epidermal inducer belonging to the TGF‐β family.^19^, ^20^, ^21^ BMP2 and BMP7 share functional similarities with BMP4 during embryonic development and in dissociated cells.^22^ In chick embryos, BMP signalling directs epidermal fate in the presence of WNT, which blocks neural differentiation by inhibiting FGF signalling (Figure 1C).^23^, ^24^ Similar mechanisms have been observed during ectoderm formation in *Xenopus*.^25^ TFAP2C and p63, the master transcriptional regulators of epidermal commitment, are under the control of BMP signalling and drive epidermis initiation.^26^, ^27^, ^28^, ^29^ Besides functioning as epidermal inducers, BMPs have been implicated in the subsequent epidermal stratification process. For instance, BMP4 accelerates the growth of basal progenitor cells by activating the downstream FGF7 and FGF10 pathways and restoring the abnormal spinous layer caused by the disruption of WNT ligands.^30^ High levels of BMP6 have been observed in the suprabasal layers of the murine epidermis, which persist until day six postpartum. Transgenic mice overexpressing BMP6 exhibit aberrant skin development.^31^, ^32^, ^33^ These studies highlight the decisive role of BMPs in epidermal cell proliferation and differentiation. In addition, other TGF‐β family members, such as TGF‐β1 and TGFβRs, are also involved in the development of the human foetal epidermis, while their role in adult epidermal development requires further investigation.^34^, ^35^

Besides TGF‐β family proteins, FGFs have been implicated in embryonic skin development. FGF signalling acts as an antagonist of BMP signalling to drive neural rather than epidermal fate (Figure 1C).^23^ However, evidence suggests that some FGF family proteins and their receptors can act as keratinocyte mitogens, either independently or synergistically with BMP signalling to promote epidermal stratification.^23^, ^36^ For example, FGF7 and FGF10 can be activated by BMP4 and together they can regulate basal cell proliferation through the Smad1/5/8 pathway, thereby promoting epidermal stratification.^30^ Correspondingly, FGF10‐deficient mice exhibit abnormal skin development due to insufficient basal cells that are required for a normal stratified epidermis, confirming the role of FGF10 in cell proliferation.^37^, ^38^, ^39^ This phenomenon has also been observed in *Fgfr2‐IIIb*‐null mice.^39^

## 4 TGF‐β AND FGF SIGNALLING IN THE EMBRYOGENESIS OF THE DERMIS

Unlike the epidermis, the dermis develops from both the ectoderm as well as mesoderm depending on its distinct location in the embryo. Various studies have been conducted to investigate the genetic programme of distinct dermis development at different embryonic stages.

### 4.1 TGF‐β and FGF signalling in craniofacial dermis development

During craniofacial development, NCCs are the main contributors to dermis formation. Due to their stem‐like multi‐potent characteristics and extensive migration potential, NCCs are considered as the fourth germ layer. In embryos, neural crest (NC) formation is promoted by programmed morphological changes and mediated by contact signals from adjacent tissues, such as the neural ectoderm, non‐neural ectoderm and mesoderm.^40^ First, the neural ectodermal fate is specified from the ectoderm under the influence of the notochord, a flexible rod‐like structure derived from the mesoderm. As the neural ectoderm thickens and flattens, a neural plate forms that give rise to the neural tube. During this process, NCCs appear as recognisable neuroepithelial cells at the border of the neural plate. Subsequently, these cells undergo epithelial‐to‐mesenchymal transition (EMT) and migrate to diverse destinations to generate special tissues or cell types, including the craniofacial dermis.^41^, ^42^ However, some studies have claimed that NC induction occurs prior to the development of the mesoderm, neural ectoderm and neural plate because of its contribution to the ectodermal and mesenchymal structures. The exact induction time of NC is also debated.

**FIGURE 2:**
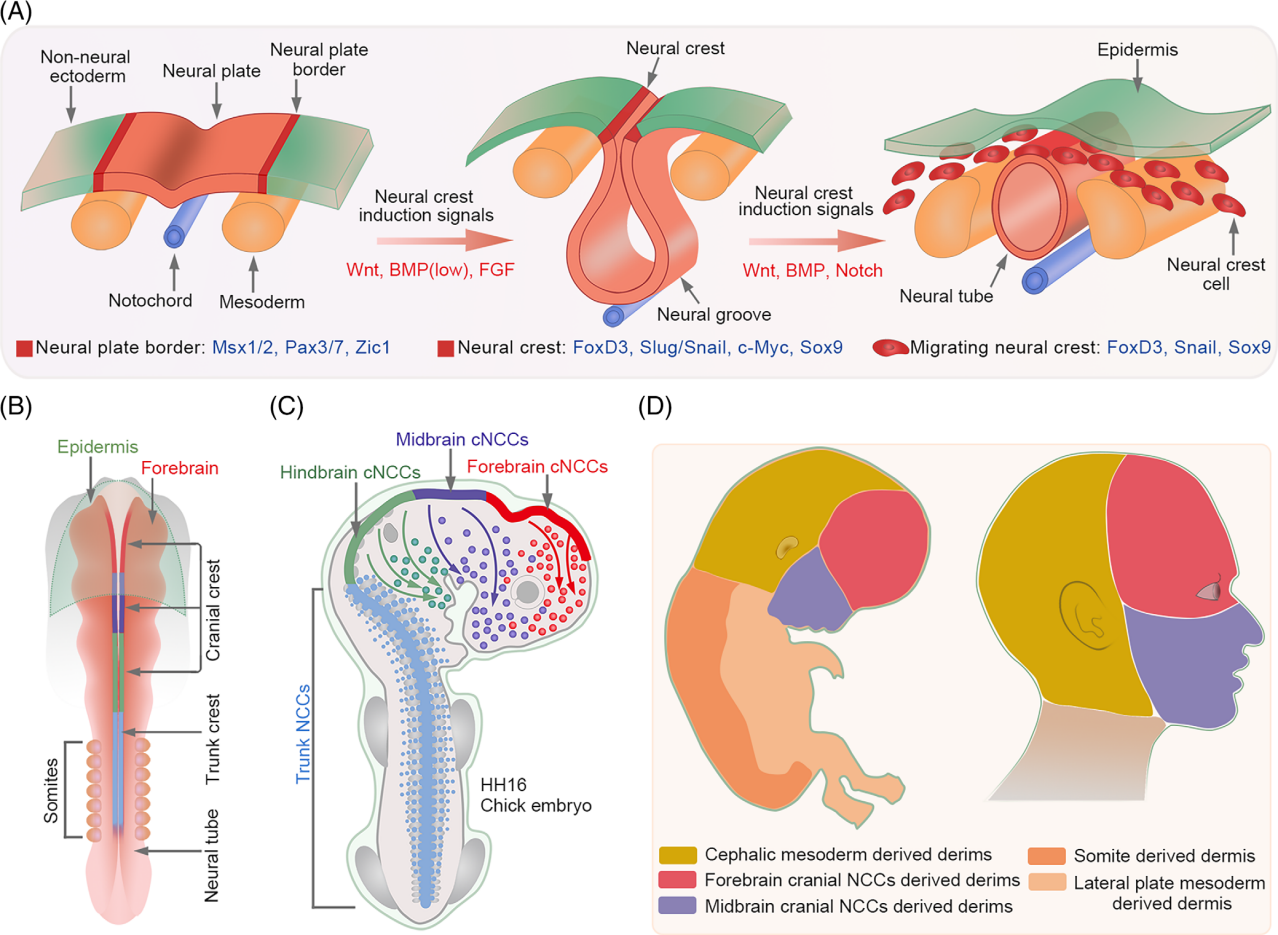
The development and migration of the neural crest cells (NCCs). (A) Neural crest (NC) formation and NCC migration during embryonic development. NC regionalization at the boundary of the neural plate and epidermis is a multi‐step process. First, the border of the neural plate is set by neural plate inductive signals (fibroblast growth factor, bone morphogenetic protein [BMPs] and WNT) secreted from the ventral ectoderm and paraxial mesoderm. These secreted factors induce the expression of border regionalization genes such as *Msx1/2*, *Pax3/7* and *Zic1*. Subsequently, the NC is specified with the expression of FoxD3, Slug/Snail, c‐Myc, SOX9 and Id in the border cells, which inhibits this region from becoming either the neural plate or epidermal tissue. Delamination and migration of NCCs are primarily induced by FoxD3, Snail and SOX9. (B) Overview of different NC subpopulations that are present along the anteroposterior axis in E8.0 (six somites) mouse embryos. The cranial NCCs are formed between the forebrain and the sixth rhombomere (R6) of the hindbrain. (C) In HH16 chick embryos, the forebrain and midbrain cNCCs migrate from the neural plate border to the frontonasal mass. HH, Hamburger and Hamilton developmental stages. (D) The development and various origins of the human craniofacial dermis. (Left) Different colours highlight regions with dermis of different origins. (Right) Regions of the developing and adult face that correspond to different NC populations present along the posterior axis.

BMPs are regarded as major factors regulating epidermal development. Recent studies have demonstrated that BMPs are required for NC induction.^43^, ^44^, ^45^ In chick embryos, BMP4 has been implicated in the formation of the dorsal neural tube, where it functions as a dorsalizing agent that mediates the differentiation of dorsal cell types.^46^ The inactivation of BMP4 during neurulation results in the suppression of NC formation.^47^ In addition, BMPs control NC migration by regulating the downstream target RhoB, a GTP‐binding protein. Delamination of NC from the neural tube is prevented by the reduced expression of RhoB and cadherin‐6B due to the C3 exotoxin or Noggin.^48^, ^49^ Further evidence for the role of BMP signalling in NC development came from studies on its relationship with EMT and cell proliferation, both of which are associated with NC migration.^50^ In mice, BMP2 rather than BMP4 appears to be responsible for this effect.^51^, ^52^ Disrupting the BMP signalling in specific areas populated with pre‐migratory and migratory NCCs results in the ablation of migratory cranial NCCs and a subsequent deficiency of cranial NC derivatives in the target region.^53^ Collectively, these studies support the crucial role of BMP in modulating NC formation (Figure 2A).

Besides BMPs, FGF signalling is also involved in NC induction, but at the gastrula stage. Experiments in chick embryos provided the first in vivo evidence that FGF signalling participates in NC induction at the early gastrula stages by modulating its downstream MAPK signalling and inhibiting BMP4/Smad expression. Consistently, the inhibition of FGF/MAPK activity at this stage results in reduced expression of Pax7, which is required for NC induction. During neurulation, a stage following gastrulation, Pax7 colocalizes with Smad1/5/8 rather than FGF/MAPK, suggesting that the FGF and BMP pathways are required for the early and later development of NC, respectively.^54^ This phenomenon has also been observed in *Xenopus laevis* embryos. During gastrulation, low BMP levels are needed for the formation of neural borders. At later stages, Fhl3, a scaffold LIM domain protein, activates BMP and BMP‐dependent WNT signalling pathways, which further promotes NC specification.^55^ FGF signalling, together with BMP and WNT signalling can also induce NC formation even after non‐neural ectodermal fate specification (Figure 2A).^56^ Treatment with FGFs, BMP4 and WNT modulators can differentiate human pluripotent stem cells into cranial NCCs expressing AP2α, MSX1 and DLX1.^57^ Although FGF and BMP signalling pathways are crucial for the establishment of NC, the levels of FGF and BMP signalling proteins are also crucial for cell fate determination. For example, low BMP expression promotes early induction of NC, whereas high expression favours epidermal fate. Similarly, moderate FGF activity induces NC, whereas high activity promotes the generation of neural progenitor cells. Therefore, effective cooperation and appropriate activity of these signalling pathways are required for NC establishment.

Besides their role in NC induction, in vitro experiments have shown that FGF2 and FGF8 possess chemotactic activity against mouse mesencephalic NCCs. Supplementation with anti‐FGF2 or anti‐FGF8 antibodies can inhibit this effect. In organ culture systems (explants), FGF8 indirectly affects the chemotactic migration of NC by regulating the FGF2 distribution pattern.^58^ However, in a conditional FGF8 knockout mouse model developed by Trumpp et al., FGF8 depletion does not influence NC formation but promotes NC maintenance and survival.^59^, ^60^, ^61^

Generally, NCCs are formed along the entire anterior–posterior axis of the developing mammalian embryo. NCCs can be classified into distinct subpopulations (cranial, vagal, trunk and sacral NCCs) based on their axial location and origin (Figure 2B). NCCs display varied migratory properties and contribute to the formation of multiple structures. Numerous studies have been performed to elucidate the mechanism underlying NC formation. However, very few have focused on its further differentiation into the dermis.

The avian craniofacial dermis is derived entirely from the cranial NCCs,^62^ while in mammals, the facial dermis is derived from cranial NCCs, and the cranial dermis may be predominantly derived from the paraxial/cephalic mesoderm.^63^, ^64^, ^65^ In E8.5‐E9.5 mice embryos or HH16 chick embryos, forebrain and midbrain cranial NCCs migrate dramatically from the neural plate border to the frontonasal mass.^66^, ^67^ These migrating forebrain and midbrain cranial NCCs give rise to fibroblast precursors of the forehead and face skin, respectively, in the anterior supraorbital arch (SOA) region (Figure 2C).^68^, ^69^ Subsequently, cells of the cephalic mesoderm migrate to the posterior SOA region by E10.5 and give rise to dermal fibroblast precursors for most of the cranial skin. From E10.5, specific cranial dermal fibroblast progenitors expand apically from the SOA to fill the cranial region.^68^ The WNT/β‐catenin signalling plays a predominant role in promoting the dermal cell fate of cephalic mesoderm cells via its target Dermo1.^70^ Based on these studies, it can be concluded that the human craniofacial dermis originates from different cranial NCC subpopulations and cephalic mesodermal cells (Figure 2D).

**FIGURE 3:**
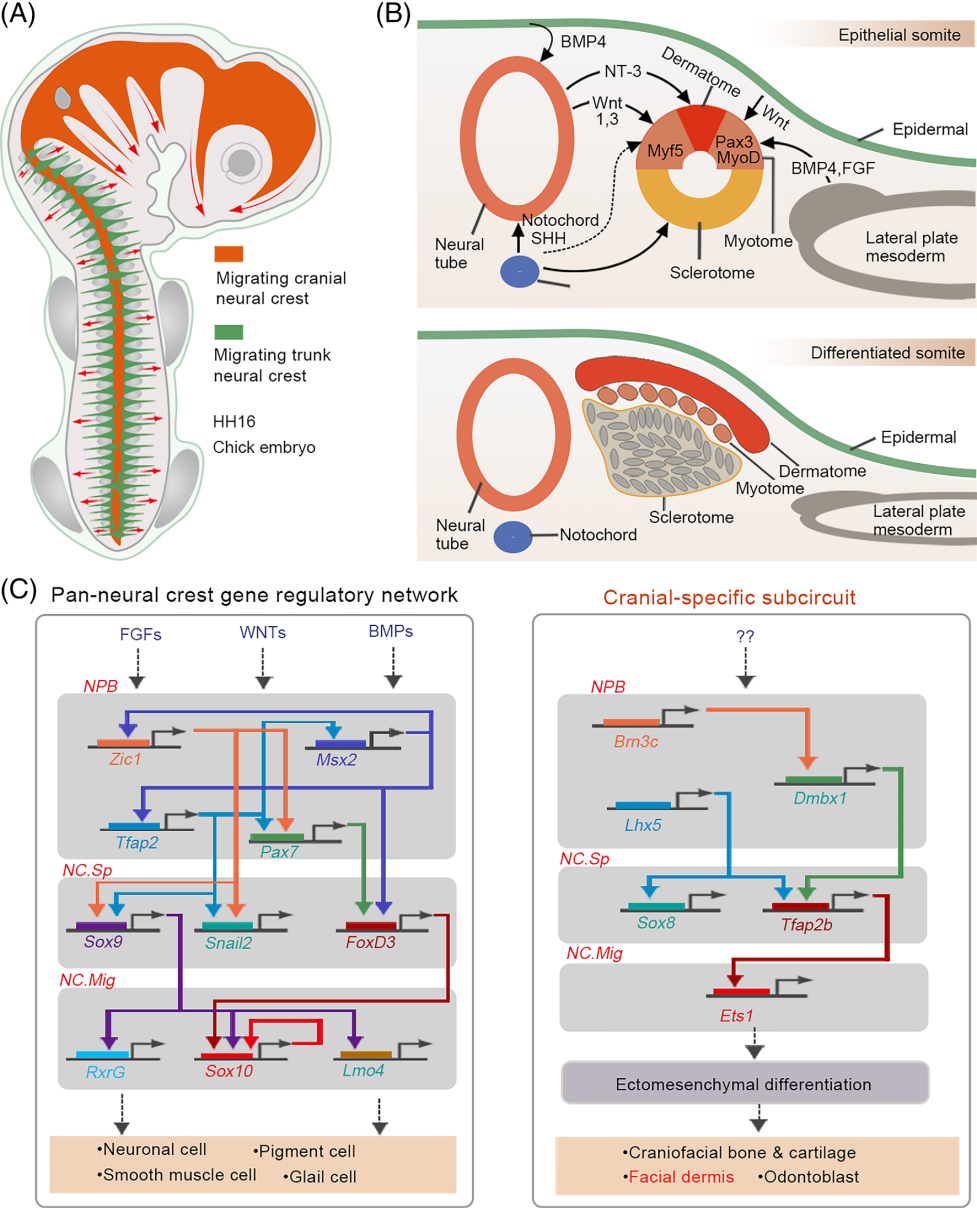
The differentiation of neural crest cells (NCCs). (A) Migration pathway of different NC subpopulations along the anteroposterior axis. (B) Model of major postulated interactions during the patterning of the somites (upper). Dermatome, myotome and sclerotome are generated from somites (lower). (C) Axial‐specific features of the NC gene regulatory network (Left). Simplified pan‐NC gene regulatory network, which does not account for differences among subpopulations, and the cranial‐specific subcircuit contributing to ectomesenchymal differentiation (Right). The cranial NCCs that give rise to the craniofacial skeleton and facial dermis are the only subpopulation possessing ectomesenchymal potential in amniotes. NPB, neural plate border; NC Sp, neural crest specification; NC. Mig, neural crest migration; NT‐3, neurotrophin‐3.

Comparative transcriptional profiling of cranial and trunk NC cell populations revealed hundreds of cranial‐ and trunk‐enriched genes.^71^ Functional and biochemical analyses revealed that a group of transcription factors enriched in cranial NCCs promotes the delineation of a cranial‐specific regulatory sub‐circuit (Figure 3). Notably, ectopic expression of three components (Ets1, SOX8 and Tfap2b) of this circuit in the trunk NCCs is sufficient to reprogram these cells into progenitors with “cranial‐like” identity,^71^ indicating the importance of axial‐specific genetic programmes in cell fate determination within the NC.

### 4.2 Role of TGF‐β and FGF signalling in the dorsal and ventral dermis

The dorsal and ventral dermis differ from the craniofacial dermis and have different developmental origins. The dorsal dermis develops from the paraxial mesoderm that lies along the notochord. After the segmentation of the paraxial mesoderm, somites are formed and are further subdivided into dermomyotomes, myotomes and sclerotomes, among which dermomyotomes contribute to the dorsal dermis. The lateral plate mesoderm, which is separated from the adjacent paraxial mesoderm by a narrow region of intermediate mesoderm, gives rise to the dermis of the ventral and flank regions.^72^

Previous studies showed that TGF‐β, BMP and FGF signalling pathways are involved in the development of the dermis from the mesoderm. Smad2 and Smad3, the downstream intracellular effectors of the TGF‐β pathway, cooperatively regulate mesoderm formation in mice embryos. Once inhibited, the specification of axial and paraxial mesodermal derivatives is also disrupted.^73^ Compared to other TGF‐β family members, BMPs possess more remarkable characteristics and perform diverse functions. BMPs not only regulate the fate choice between the paraxial and lateral mesoderm but also act as regulators of somitogenesis and dermomyotome development. In early chick embryos, BMP4 is expressed in the neural tube. As the development progresses, its expression extends to the lateral plate mesoderm along the anteroposterior axis.^74^, ^75^ Noggin, an antagonist of BMP4, is mainly expressed in presumptive somites that develop from the paraxial mesoderm.^76^ Implantation of BMP4‐expressing cells into the presomitic mesoderm (PSM) disrupts medial somite formation, and cells of this region acquire the properties of lateral plate mesoderm. Furthermore, it has been observed that the dermomyotomes are not formed in the presence of BMPs, indicating the negative regulation of BMP signalling in paraxial mesoderm and somitogenesis.^74^, ^77^ Accordingly, noggin administration to the presumptive lateral plate induces the formation of somites in the original lateral plate region.^76^ At the same time, blocking BMP signalling in the lateral plate also results in the loss of expression of genes related to the lateral plate mesoderm and limb formation, which subsequently affects lateral plate mesoderm specification and limb bud mesenchyme formation.^78^ Bidirectional studies have collaboratively illustrated the distinct roles of BMP4 in paraxial mesoderm and lateral plate mesoderm development. In addition, BMP4 functions as a lateralizer and inducer of the lateral mesoderm specification. However, BMP inhibition is required for paraxial mesoderm formation and differentiation.

Unlike BMP4, which functions in the paraxial mesoderm, FGFs (FGF4 and FGF8) promote the specification of the PSM and its further segmentation into somites.^79^, ^80^, ^81^ Besides BMP4 and FGFs, a combination of WNTs (WNT1 and WNT3a), which are induced by BMP4 from the dorsal neural tube and low levels of sonic hedgehog from the notochord and floor plate are also necessary for further differentiation of paraxial mesoderm into the epaxial myotome. Appropriate levels of neurotrophin‐3 can also promote dermatome specification, which generates the dermis and mesenchymal connective tissue of the back skin (Figure 3B,C).^82^

In conclusion, the dermis of the mammalian skin has multiple embryonic origins. Head and facial fibroblasts are derived from the NC or paraxial/cephalic mesoderm, whereas dorsal and ventral trunk dermal fibroblasts are derived from the somites and lateral plate mesoderm, respectively.

## 5 ROLE OF TGF‐β AND FGF SIGNALLING IN HF DEVELOPMENT

**FIGURE 4:**
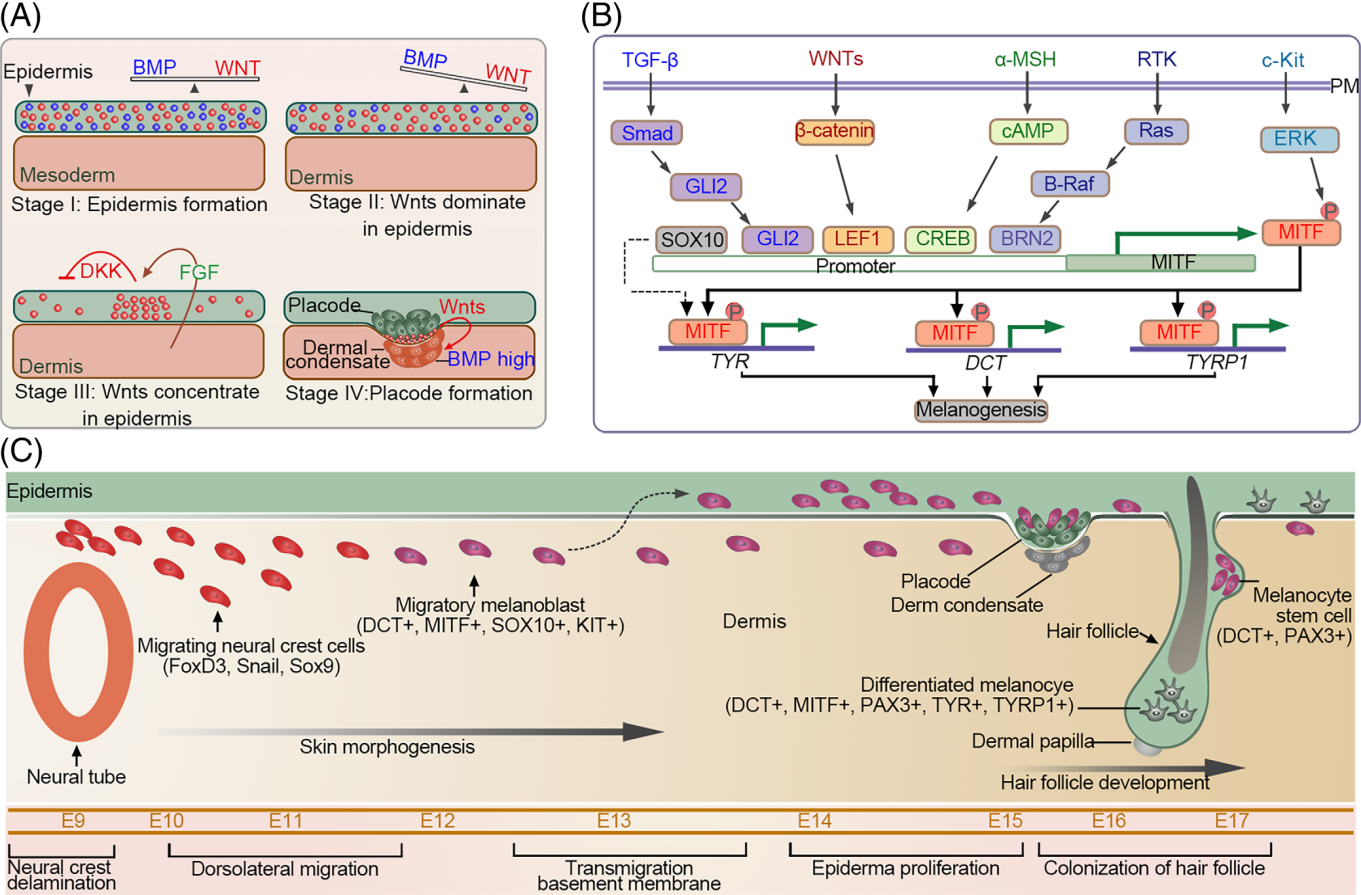
Illustration showing hair follicle (HF) formation and pigmentation. (A) The mechanism of hair placode formation and dermal condensation. Stage I: During gastrulation, the epidermis is formed under the influence of a high concentration of both bone morphogenetic proteins (BMPs) and WNTs. Stage II: With the development of the embryo, the concentration of BMPs is gradually reduced, which results in a relatively high level of WNT signalling. Stage III: fibroblast growth factors, WNTs and DKKs interact with each other leading to the nonuniform distribution of the WNT signalling in the epidermis. Stage IV: Placodes are formed in the epidermal region with a high level of WNT signalling. WNTs secreted from the placode induce dermal condensation. (B) Key molecules and signalling pathways implicated in melanocyte homeostasis and melanogenesis. Several transcription factors, including Gli2, SOX10, cAMP response element‐binding protein (CREB), Lef1 and microphthalmia‐associated transcription factor (MITF) play crucial roles in regulating melanocyte proliferation, differentiation and survival. MITF is the master regulator of melanocyte development and its expression is regulated by the synergistic action of Gli2, SOX10, Lef1 and CREB on the MITF promoter. Once translated, MITF protein is phosphorylated by the Erk kinase and then it promotes the downstream activation of c‐Kit, the stem cell factor receptor tyrosine kinase. Phosphorylation of MITF results in the stabilisation of the MITF‐p300 transactivation complex, which enhances its transcriptional activity to induce the expression of target genes, including *Dct*, *Typ* and *Tyrp1*. These genes encode melanogenic enzymes that participate in eumelanin and pheomelanin synthesis within melanosomes. (C) Schematic overview of different stages of melanoblast development in mouse embryos.

The HFs are skin appendages that develop from the ectoderm. In mice, the primary HF structure, placode, is formed at approximately E12.5–14.5 in response to the WNT signals from the underlying dermis. The placode then releases signals to recruit surrounding mesenchymal cells and generate dermal condensate (DC), the precursor of the dermal papilla (DP) (Figure 4A).^83^, ^84^ Subsequently, a second dermal signal is sent to the placode from the DC that induces downward growth of the HF, resulting in the encapsulation of DC by epithelial cells.^84^ Repeated epithelium–mesenchymal signal exchanges lead to the generation of multiple HF cell lineages and the maturation of HF structures. Unlike other organs, the developed HF has remarkable potential for self‐renewal. HF periodically regenerates by undergoing repeated cycles of anagen, catagen and telogen phases. This complex process involves precise molecular regulation, accurate spatiotemporal communication, cooperation of several factors and competition.

During HF development, TGF‐β2 reportedly acts as a promoter that induces the migration of the mesenchymal cells towards the placode.^85^ In contrast to the TGF‐β signalling, the presence of BMPs significantly impacts the placode and DC formation.^85^ During HF development, BMPs collaborate with WNT signalling to regulate HF distribution patterns.^86^ In mice, sustained β‐catenin expression in the epidermis contributes to high HF density. Moreover, WNT/β‐catenin signalling increases the downstream BMP activity, which modulates hair spacing by inhibiting the neighbouring placode formation, thus, establishing appropriate HF patterning.^87^, ^88^, ^89^ Unlike WNT and BMP signalling pathways, FGF signalling plays a more complex role in HF induction, depending on the FGF members involved. In mice, Fgfr2‐IIIb deficiency results in the loss of placode markers and abnormal hair formation.^39^ Similarly, the ablation of FGF20 also leads to the loss of HFs due to the absence of DC.^90^, ^91^, ^92^ In cultured DP, supplementation with FGF9 upregulates representative DP biomarkers and increases HF size when these DPs are transplanted into mice along with keratinocytes.^93^ Collectively, these studies suggest that some FGFs positively regulate HF formation. However, the administration of FGF7 suppresses hair development and decreases HF size,^93^, ^94^ indicating both the negative and positive roles of FGFs in HF induction.

## 6 TGF‐β AND FGF SIGNALLING PATHWAYS IN SKIN PIGMENTATION

Melanocyte stem cells (McSCs) and melanocytes are responsible for skin pigmentation. It is widely accepted that most melanocytes in the skin are originally derived from the trunk NCCs.^95^, ^96^ Irrespective of the animal species, SOX10‐expressing melanoblast‐glial bipotent progenitor cells formed in the trunk region are generally considered as immediate precursors of melanoblast and neurogenic populations. Neural progenitor cells migrate further via the ventral route, whereas melanocyte progenitor cells migrate dorsolaterally and terminally differentiate into melanocytes.^97^

Melanocytes produce melanin and distribute it to surrounding keratinocytes.^98^ Factors derived from the keratinocytes and dermal fibroblasts regulate this process. TGF‐β secreted by these cells maintains immature McSCs by downregulating the expression of microphthalmia‐associated transcription factor (MITF) and its downstream melanogenic genes in melanoblasts/melanocytes.^99^ In the absence of TGF‐β signalling, hair greying is accelerated (Figure 4B,C).^100^, ^101^

Unlike TGF‐β, BMPs differentially regulate skin pigmentation depending on the type of BMP.^102^ BMP2 stimulates melanogenesis by upregulating tyrosinase expression in differentiated melanocytes.^103^ BMP6 not only enhances melanogenesis by promoting the expression and activity of tyrosinase but also promotes melanin transfer from melanocytes to keratinocytes. In contrast to BMP6, BMP4 inhibits melanin production and transfer.^104^ BMPs are also involved in suppressing the differentiation of NC cells into melanocytes.^99^ Nicole et al. demonstrated that BMPs collaborate with the WNT pathway to promote stem cell commitment by facilitating the LEF1‐ and MITF‐dependent differentiation of McSCs.

In contrast to the TGF‐β effect on melanocytes, FGF signalling is capable of promoting melanin synthesis by upregulating melanocyte proliferation. FGF2 in cooperation with cAMP stimulators induces melanocyte proliferation.^105^ In *Ciona intestinalis*, FGF signalling regulates central nervous system development and pigment cell formation. FGF signalling participates in the early pigment cell type‐specific regulation of several components of melanosome vesicular transport.^106^ The FGF/WNT signalling crosstalk is also involved in the induction and differentiation of *C*. *intestinalis* pigment cells. During the late gastrula stage, FGF/MAPK/Ets signalling sensitises pigment cell precursors to WNT signals by directly controlling the WNT downstream Ci‐Tcf transcription.^107^

## 7 CONCLUSIONS AND FUTURE PERSPECTIVES

In this review, we described the role of TGF‐β and FGF signalling in the epidermal determination that is important for epidermis development, NC maintenance during neurulation that is required for the craniofacial dermis development, paraxial mesoderm or lateral plate mesoderm fate decision that is important for dorsal or ventral dermis formation. FGFs not only cooperate with BMPs to modulate these processes but also act as the major factors regulating NC induction and somite segmentation, processes that are required for the development of the dorsal dermis. Besides, FGFs and BMPs also promote cell survival and proliferation.

Although extensive studies have been performed, several questions on mechanisms underlying skin development remained unanswered.

First, during dermis development, the exact levels of FGFs and BMPs that are crucial for cell fate decision are not clearly understood.

Second, although the origins of the dermis of different body regions have been identified, the underlying mechanisms remain unclear. For example, the regulatory network underlying the development of the craniofacial dermis from the NC and the dorsal dermis from the dermomyotome remains largely unknown.

Finally, the role of other signalling pathways that cooperate with the TGF‐β and FGF signalling pathways during skin development remains poorly understood.

Due to ethical issues and technological hurdles, it is difficult to investigate the complete mechanism of embryonic skin development. Hence, cultured skin or HFs, especially skin organoids, are the most suitable models that can be used to address these issues. However, it is necessary to understand the regulatory networks and determine the identity of the in vitro generated skin/HFs.

To summarise, a better understanding of the functional role of TGF‐β and FGF signalling pathways in skin development will provide new perspectives on skin repair and regeneration. This information can be exploited for both basic research as well as clinical applications. Further studies are required to elucidate the detailed regulatory mechanisms underlying skin development, repair, and regeneration.

## AUTHOR CONTRIBUTIONS

Xusheng Wang provided original ideas, designed the study and supervised the study. Xinxin Li, Rongfang Xie and Yilin Luo wrote the initial version of the manuscript. Runlu Shi prepared the figures. Yuanqiang Ling and Xiaojing Zhao collected the data. Xuejuan Xu and Weiwei Chu revised the manuscript and designed the study.

## FUNDING INFORMATION

This work was supported by the Shenzhen Technology and Innovation Commission (JCYJ20200109142444449, JCYJ20210324120007021 and JCYJ20180307123901314), National Natural Science Foundation of China (31801196 and 82102526), Guangdong Basic and Applied Basic Research Foundation (2019A1515110463), and the Foshan 14th‐15th high‐level key specialty construction project.

## CONFLICT OF INTEREST STATEMENT

The authors declare that they have no competing interests.

## Data Availability

Data sharing is not applicable to this article as no new data were generated or analyzed in this study.
